# *N*-(3-Chloro­phen­yl)-2-nitro­benzene­sulfonamide

**DOI:** 10.1107/S1600536812033272

**Published:** 2012-07-28

**Authors:** U. Chaithanya, Sabine Foro, B. Thimme Gowda

**Affiliations:** a Department of Chemistry, Mangalore University, Mangalagangotri 574 199, Mangalore, India; b Institute of Materials Science, Darmstadt University of Technology, Petersenstrasse 23, D-64287 Darmstadt, Germany

## Abstract

In the title compound, C_12_H_9_ClN_2_O_4_S, the dihedral angle between the aromatic rings is 73.65 (7)°. The amide H atom shows bifurcated hydrogen bonding, generating an intra­molecular *S*(7) and an inter­molecular *C*(4) motif.

## Related literature

For studies on the effects of substituents on the structures and other aspects of *N*-(ar­yl)-amides, see: Alkan *et al.* (2011 ▶); Bowes *et al.* (2003 ▶); Gowda *et al.* (2000 ▶); Saeed *et al.* (2010 ▶); Shahwar *et al.* (2012 ▶), of *N*-aroylsulfonamides, see: Suchetan *et al.* (2012 ▶), of *N*-aryl­sulfonamides, see: Gowda *et al.* (2002 ▶) and of *N*-chloro­aryl­sulfonamides, see: Gowda & Shetty (2004 ▶); Shetty & Gowda (2004 ▶). For hydrogen-bonding patterns and motifs, see: Adsmond *et al.* (2001 ▶); Allen *et al.* (1998 ▶); Bernstein *et al.* (1995 ▶); Etter (1990 ▶).
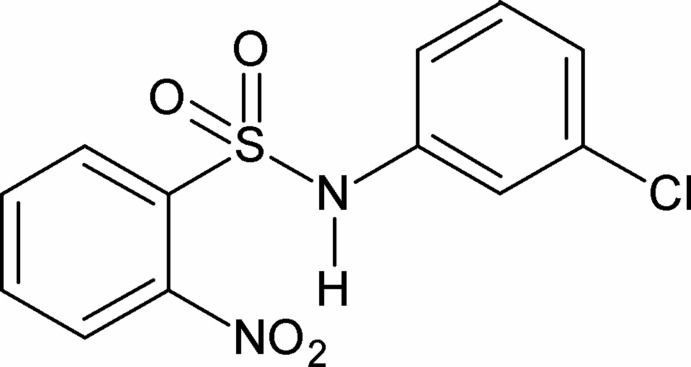


## Experimental

### 

#### Crystal data

C_12_H_9_ClN_2_O_4_S
*M* *_r_* = 312.72
Monoclinic, 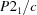

*a* = 8.9856 (7) Å
*b* = 9.5794 (8) Å
*c* = 15.796 (1) Å
β = 103.120 (8)°
*V* = 1324.17 (17) Å^3^
*Z* = 4
Mo *K*α radiation
μ = 0.46 mm^−1^
*T* = 293 K
0.48 × 0.48 × 0.24 mm

#### Data collection

Oxford Diffraction Xcalibur diffractometer with a Sapphire CCD detector
Absorption correction: multi-scan (*CrysAlis RED*; Oxford Diffraction, 2009 ▶) *T*_min_ = 0.809, *T*_max_ = 0.898
5230 measured reflections
2702 independent reflections
2201 reflections with *I* > 2σ(*I*)
*R*_int_ = 0.013

#### Refinement

*R*[*F* ^2^ > 2σ(*F* ^2^)] = 0.039
*wR*(*F* ^2^) = 0.100
*S* = 1.03
2702 reflections
184 parameters
1 restraint
H atoms treated by a mixture of independent and constrained refinement
Δρ_max_ = 0.39 e Å^−3^
Δρ_min_ = −0.51 e Å^−3^

### 

Data collection: *CrysAlis CCD* (Oxford Diffraction, 2009 ▶); cell refinement: *CrysAlis CCD*; data reduction: *CrysAlis RED* (Oxford Diffraction, 2009 ▶); program(s) used to solve structure: *SHELXS97* (Sheldrick, 2008 ▶); program(s) used to refine structure: *SHELXL97* (Sheldrick, 2008 ▶); molecular graphics: *PLATON* (Spek, 2009 ▶); software used to prepare material for publication: *SHELXL97*.

## Supplementary Material

Crystal structure: contains datablock(s) I, global. DOI: 10.1107/S1600536812033272/bt5982sup1.cif

Structure factors: contains datablock(s) I. DOI: 10.1107/S1600536812033272/bt5982Isup2.hkl

Supplementary material file. DOI: 10.1107/S1600536812033272/bt5982Isup3.cml

Additional supplementary materials:  crystallographic information; 3D view; checkCIF report

## Figures and Tables

**Table 1:** Hydrogen-bond geometry (Å, °)

*D*—H⋯*A*	*D*—H	H⋯*A*	*D*⋯*A*	*D*—H⋯*A*
N1—H1*N*⋯O3	0.82 (2)	2.33 (2)	2.864 (2)	123 (2)
N1—H1*N*⋯O2^i^	0.82 (2)	2.33 (2)	3.061 (2)	148 (2)

Symmetry code: (i) 
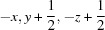
.

**Fig. 1.**
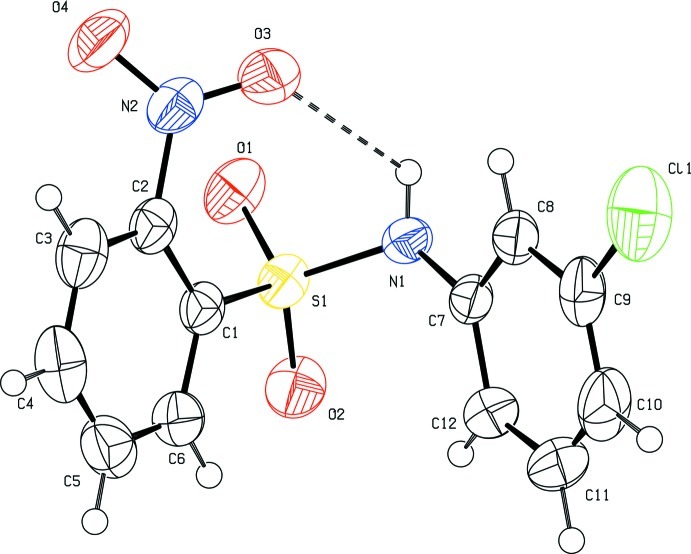
Molecular structure of the title compound, showing the atom labelling scheme and with displacement ellipsoids drawn at the 50% probability level.

**Fig. 2.**
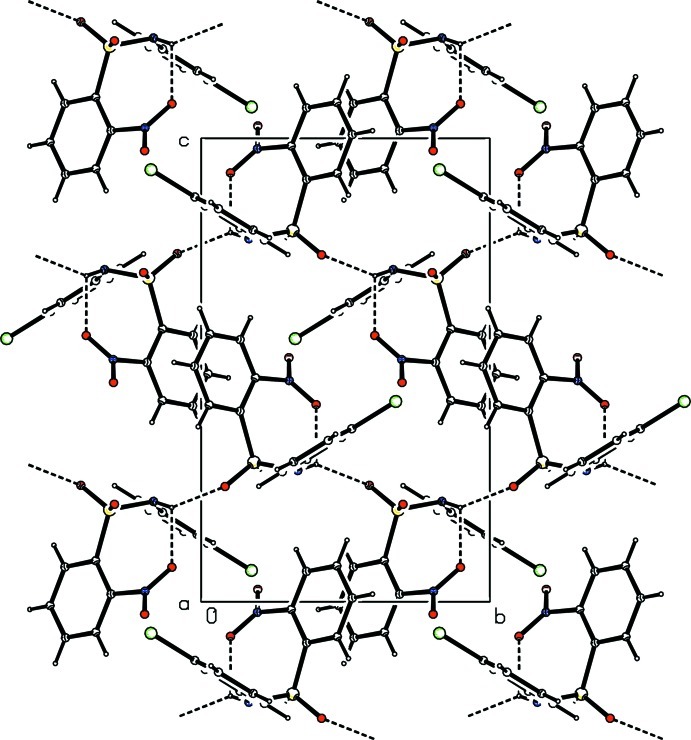
Molecular packing of the title compound with hydrogen bonding shown as dashed lines.

## supplementary crystallographic information

### Comment

As part of our studies on the substituent effects on the structures and other aspects of *N*-(aryl)-amides (Alkan *et al.*, 2011; Bowes *et al.*, 2003; Gowda *et al.*, 2000; Saeed *et al.*, 2010; Shahwar *et al.*, 2012); *N*-aroylsulfonamides (Suchetan *et al.*, 2012); *N*-arylsulfonamides (Gowda *et al.*, 2002) and *N*-chloroarylsulfonamides (Gowda & Shetty, 2004; Shetty & Gowda, 2004), in the present work, the crystal structure of *N*-(3-Chlorophenyl)-2-nitrobenzenesulfonamide has been determined (Fig. 1).

The conformation of the N—C bond in the —SO_2_—NH—C segment has *gauche* torsion with respect to the S═O bonds (Fig.1), similar to that observed in *N*-(3-chlorobenzoyl)-2-nitrobenzenesulfonamide (I) (Suchetan *et al.*, 2012). Further, the conformation of the N—H bond in the —SO_2_—NH— segment is *syn* to both the *ortho*-nitro group in the sulfonyl benzene ring and *meta*-Cl atom in the anilino ring. The molecule is twisted at the S—N bond with the torsional angle of 48.46 (18)°, compared to the value of 65.41 (38)° in (I).

The dihedral angle between the sulfonyl and the anilino rings is 73.65 (7)°, compared to the value of 89.1 (1)° in (I).

The amide H-atom showed bifurcated intramolecular H-bonding with the O-atom of the *ortho*-nitro group in the sulfonyl benzene ring and the intermolecular H-bonding with the sulfonyl oxygen atom of the other molecule, generating S(7) and C(4) motifs (Adsmond *et al.*, 2001; Allen *et al.*, 1998; Bernstein *et al.*, 1995; Etter, 1990).

In the crystal, the intermolecular N–H···O (S) hydrogen bonds(Table 1) link the molecules into chains. Part of the crystal structure is shown in Fig. 2.

### Experimental

The title compound was prepared by treating 2-nitrobenzenesulfonylchloride with 3-chloroaniline in the stoichiometric ratio and boiling the reaction mixture for 15 minutes. The reaction mixture was then cooled to room temperature and added to ice cold water (100 ml). The resultant solid *N*-(3-chlorophenyl)-2-nitrobenzenesulfonamide was filtered under suction and washed thoroughly with cold water and dilute HCl to remove the excess sulfonylchloride and aniline, respectively. It was then recrystallized to constant melting point from dilute ethanol. The purity of the compound was checked and characterized by its infrared spectra.

Rod like colourless single crystals of the title compound used in X-ray diffraction studies were grown in ethanolic solution by slow evaporation of the solvent at room temperature.

### Refinement

H atoms bonded to C were positioned with idealized geometry using a riding model with C—H = 0.93 Å. The amino H atom was freely refined with the N—H distance restrained to 0.86 (2) Å. All H atoms were refined with isotropic displacement parameters set to 1.2 *U*_eq_ of the parent atom.

### Crystal data

**Table:**

C_12_H_9_ClN_2_O_4_S	*F*(000) = 640
*M**_r_* = 312.72	*D*_x_ = 1.569 Mg m^−^^3^
Monoclinic, *P*2_1_/*c*	Mo *K*α radiation, λ = 0.71073 Å
Hall symbol: -P 2ybc	Cell parameters from 2323 reflections
*a* = 8.9856 (7) Å	θ = 2.6–27.8°
*b* = 9.5794 (8) Å	µ = 0.46 mm^−^^1^
*c* = 15.796 (1) Å	*T* = 293 K
β = 103.120 (8)°	Rod, colourless
*V* = 1324.17 (17) Å^3^	0.48 × 0.48 × 0.24 mm
*Z* = 4	

### Data collection

**Table:**

Oxford Diffraction Xcalibur diffractometer with a Sapphire CCD detector	2702 independent reflections
Radiation source: fine-focus sealed tube	2201 reflections with *I* > 2σ(*I*)
Graphite monochromator	*R*_int_ = 0.013
Rotation method data acquisition using ω scans	θ_max_ = 26.4°, θ_min_ = 2.7°
Absorption correction: multi-scan (*CrysAlis RED*; Oxford Diffraction, 2009)	*h* = −11→11
*T*_min_ = 0.809, *T*_max_ = 0.898	*k* = −11→10
5230 measured reflections	*l* = −19→15

### Refinement

**Table:**

Refinement on *F*^2^	Primary atom site location: structure-invariant direct methods
Least-squares matrix: full	Secondary atom site location: difference Fourier map
*R*[*F*^2^ > 2σ(*F*^2^)] = 0.039	Hydrogen site location: inferred from neighbouring sites
*wR*(*F*^2^) = 0.100	H atoms treated by a mixture of independent and constrained refinement
*S* = 1.03	*w* = 1/[σ^2^(*F*_o_^2^) + (0.0414*P*)^2^ + 0.7223*P*] where *P* = (*F*_o_^2^ + 2*F*_c_^2^)/3
2702 reflections	(Δ/σ)_max_ < 0.001
184 parameters	Δρ_max_ = 0.39 e Å^−^^3^
1 restraint	Δρ_min_ = −0.51 e Å^−^^3^

### Special details

**Table:**

Experimental. Absorption correction: CrysAlis RED (Oxford Diffraction, 2009) Empirical absorption correction using spherical harmonics, implemented in SCALE3 ABSPACK scaling algorithm.
Geometry. All e.s.d.'s (except the e.s.d. in the dihedral angle between two l.s. planes) are estimated using the full covariance matrix. The cell e.s.d.'s are taken into account individually in the estimation of e.s.d.'s in distances, angles and torsion angles; correlations between e.s.d.'s in cell parameters are only used when they are defined by crystal symmetry. An approximate (isotropic) treatment of cell e.s.d.'s is used for estimating e.s.d.'s involving l.s. planes.
Refinement. Refinement of *F*^2^ against ALL reflections. The weighted *R*-factor *wR* and goodness of fit *S* are based on *F*^2^, conventional *R*-factors *R* are based on *F*, with *F* set to zero for negative *F*^2^. The threshold expression of *F*^2^ > σ(*F*^2^) is used only for calculating *R*-factors(gt) *etc*. and is not relevant to the choice of reflections for refinement. *R*-factors based on *F*^2^ are statistically about twice as large as those based on *F*, and *R*- factors based on ALL data will be even larger.

### Fractional atomic coordinates and isotropic or equivalent isotropic displacement parameters (Å^2^)

**Table:**

	*x*	*y*	*z*	*U*_iso_*/*U*_eq_	
C1	0.1289 (2)	0.1345 (2)	0.41117 (13)	0.0364 (4)	
C2	0.0829 (2)	0.1878 (2)	0.48339 (13)	0.0395 (5)	
C3	0.1398 (3)	0.1350 (3)	0.56563 (14)	0.0523 (6)	
H3	0.1062	0.1705	0.6128	0.063*	
C4	0.2467 (3)	0.0298 (3)	0.57749 (17)	0.0606 (7)	
H4	0.2859	−0.0054	0.6329	0.073*	
C5	0.2954 (3)	−0.0235 (3)	0.50813 (18)	0.0578 (6)	
H5	0.3687	−0.0938	0.5165	0.069*	
C6	0.2350 (3)	0.0278 (2)	0.42487 (16)	0.0487 (5)	
H6	0.2667	−0.0105	0.3778	0.058*	
C7	0.2763 (2)	0.3656 (2)	0.30709 (12)	0.0350 (4)	
C8	0.3206 (2)	0.4862 (2)	0.35429 (13)	0.0403 (5)	
H8	0.2492	0.5418	0.3726	0.048*	
C9	0.4733 (3)	0.5219 (3)	0.37354 (15)	0.0518 (6)	
C10	0.5814 (3)	0.4401 (3)	0.34821 (19)	0.0673 (8)	
H10	0.6839	0.4657	0.3619	0.081*	
C11	0.5348 (3)	0.3200 (3)	0.3024 (2)	0.0681 (8)	
H11	0.6072	0.2635	0.2855	0.082*	
C12	0.3822 (3)	0.2810 (3)	0.28080 (16)	0.0518 (6)	
H12	0.3518	0.1998	0.2493	0.062*	
N1	0.11632 (18)	0.33458 (18)	0.28331 (11)	0.0370 (4)	
H1N	0.064 (2)	0.398 (2)	0.2966 (14)	0.044*	
N2	−0.0256 (2)	0.3049 (2)	0.47737 (12)	0.0466 (5)	
O1	−0.11286 (16)	0.19931 (18)	0.28970 (10)	0.0530 (4)	
O2	0.10397 (19)	0.08275 (17)	0.24876 (10)	0.0543 (4)	
O3	−0.02102 (19)	0.39843 (19)	0.42550 (11)	0.0568 (4)	
O4	−0.1124 (2)	0.3039 (2)	0.52670 (14)	0.0756 (6)	
Cl1	0.52921 (10)	0.67414 (9)	0.43224 (5)	0.0871 (3)	
S1	0.04801 (6)	0.18286 (6)	0.30104 (3)	0.03894 (16)	

### Atomic displacement parameters (Å^2^)

**Table:**

	*U*^11^	*U*^22^	*U*^33^	*U*^12^	*U*^13^	*U*^23^
C1	0.0351 (10)	0.0342 (10)	0.0412 (10)	−0.0101 (8)	0.0112 (8)	−0.0039 (8)
C2	0.0373 (10)	0.0400 (12)	0.0428 (11)	−0.0128 (9)	0.0122 (8)	−0.0069 (9)
C3	0.0558 (14)	0.0600 (15)	0.0418 (12)	−0.0198 (12)	0.0128 (10)	−0.0053 (11)
C4	0.0624 (16)	0.0595 (16)	0.0550 (14)	−0.0185 (13)	0.0031 (12)	0.0140 (12)
C5	0.0468 (13)	0.0469 (14)	0.0770 (18)	−0.0033 (11)	0.0087 (12)	0.0124 (13)
C6	0.0481 (12)	0.0412 (12)	0.0606 (14)	−0.0033 (10)	0.0200 (10)	−0.0003 (11)
C7	0.0323 (10)	0.0396 (11)	0.0348 (10)	0.0003 (8)	0.0110 (8)	0.0069 (8)
C8	0.0388 (11)	0.0426 (12)	0.0409 (11)	−0.0022 (9)	0.0118 (8)	0.0069 (9)
C9	0.0441 (12)	0.0574 (15)	0.0518 (13)	−0.0154 (11)	0.0068 (10)	0.0139 (11)
C10	0.0340 (12)	0.080 (2)	0.0860 (19)	−0.0089 (13)	0.0097 (12)	0.0291 (16)
C11	0.0415 (13)	0.0696 (19)	0.100 (2)	0.0161 (13)	0.0311 (14)	0.0207 (16)
C12	0.0445 (12)	0.0476 (14)	0.0681 (15)	0.0053 (10)	0.0232 (11)	0.0053 (11)
N1	0.0313 (9)	0.0385 (10)	0.0425 (9)	0.0017 (7)	0.0111 (7)	−0.0010 (7)
N2	0.0455 (10)	0.0482 (11)	0.0501 (11)	−0.0116 (9)	0.0193 (8)	−0.0181 (9)
O1	0.0340 (8)	0.0703 (11)	0.0527 (9)	−0.0150 (7)	0.0058 (7)	−0.0102 (8)
O2	0.0677 (10)	0.0496 (10)	0.0501 (9)	−0.0113 (8)	0.0227 (8)	−0.0193 (7)
O3	0.0660 (11)	0.0543 (10)	0.0548 (10)	0.0076 (8)	0.0234 (8)	−0.0031 (8)
O4	0.0798 (13)	0.0675 (12)	0.1002 (15)	−0.0106 (10)	0.0640 (12)	−0.0179 (11)
Cl1	0.0842 (5)	0.0888 (6)	0.0840 (5)	−0.0501 (4)	0.0103 (4)	−0.0107 (4)
S1	0.0383 (3)	0.0421 (3)	0.0372 (3)	−0.0093 (2)	0.0102 (2)	−0.0102 (2)

### Geometric parameters (Å, º)

**Table:**

C1—C6	1.381 (3)	C8—C9	1.379 (3)
C1—C2	1.395 (3)	C8—H8	0.9300
C1—S1	1.788 (2)	C9—C10	1.376 (4)
C2—C3	1.380 (3)	C9—Cl1	1.740 (3)
C2—N2	1.475 (3)	C10—C11	1.374 (4)
C3—C4	1.376 (4)	C10—H10	0.9300
C3—H3	0.9300	C11—C12	1.387 (3)
C4—C5	1.368 (4)	C11—H11	0.9300
C4—H4	0.9300	C12—H12	0.9300
C5—C6	1.393 (3)	N1—S1	1.6265 (18)
C5—H5	0.9300	N1—H1N	0.823 (16)
C6—H6	0.9300	N2—O3	1.221 (2)
C7—C8	1.383 (3)	N2—O4	1.222 (2)
C7—C12	1.384 (3)	O1—S1	1.4243 (15)
C7—N1	1.432 (2)	O2—S1	1.4294 (16)
			
C6—C1—C2	117.7 (2)	C10—C9—C8	121.8 (2)
C6—C1—S1	117.27 (16)	C10—C9—Cl1	119.61 (19)
C2—C1—S1	124.76 (16)	C8—C9—Cl1	118.6 (2)
C3—C2—C1	121.3 (2)	C11—C10—C9	118.6 (2)
C3—C2—N2	116.0 (2)	C11—C10—H10	120.7
C1—C2—N2	122.67 (19)	C9—C10—H10	120.7
C4—C3—C2	119.7 (2)	C10—C11—C12	121.4 (2)
C4—C3—H3	120.2	C10—C11—H11	119.3
C2—C3—H3	120.2	C12—C11—H11	119.3
C5—C4—C3	120.3 (2)	C7—C12—C11	118.5 (2)
C5—C4—H4	119.8	C7—C12—H12	120.7
C3—C4—H4	119.8	C11—C12—H12	120.7
C4—C5—C6	119.8 (2)	C7—N1—S1	122.41 (14)
C4—C5—H5	120.1	C7—N1—H1N	112.1 (16)
C6—C5—H5	120.1	S1—N1—H1N	110.8 (16)
C1—C6—C5	121.1 (2)	O3—N2—O4	123.9 (2)
C1—C6—H6	119.5	O3—N2—C2	118.63 (17)
C5—C6—H6	119.5	O4—N2—C2	117.5 (2)
C8—C7—C12	121.12 (19)	O1—S1—O2	118.79 (10)
C8—C7—N1	117.65 (18)	O1—S1—N1	106.94 (10)
C12—C7—N1	121.2 (2)	O2—S1—N1	107.70 (9)
C9—C8—C7	118.5 (2)	O1—S1—C1	109.15 (9)
C9—C8—H8	120.8	O2—S1—C1	105.64 (10)
C7—C8—H8	120.8	N1—S1—C1	108.25 (9)
			
C6—C1—C2—C3	−0.7 (3)	C8—C7—C12—C11	−0.3 (3)
S1—C1—C2—C3	173.26 (16)	N1—C7—C12—C11	177.0 (2)
C6—C1—C2—N2	177.83 (18)	C10—C11—C12—C7	−0.5 (4)
S1—C1—C2—N2	−8.2 (3)	C8—C7—N1—S1	−129.76 (17)
C1—C2—C3—C4	1.4 (3)	C12—C7—N1—S1	52.8 (2)
N2—C2—C3—C4	−177.20 (19)	C3—C2—N2—O3	142.9 (2)
C2—C3—C4—C5	−0.6 (3)	C1—C2—N2—O3	−35.7 (3)
C3—C4—C5—C6	−0.9 (4)	C3—C2—N2—O4	−35.2 (3)
C2—C1—C6—C5	−0.8 (3)	C1—C2—N2—O4	146.2 (2)
S1—C1—C6—C5	−175.25 (17)	C7—N1—S1—O1	165.95 (15)
C4—C5—C6—C1	1.6 (3)	C7—N1—S1—O2	−65.32 (17)
C12—C7—C8—C9	1.1 (3)	C7—N1—S1—C1	48.46 (18)
N1—C7—C8—C9	−176.38 (18)	C6—C1—S1—O1	135.23 (16)
C7—C8—C9—C10	−1.0 (3)	C2—C1—S1—O1	−38.75 (19)
C7—C8—C9—Cl1	179.54 (15)	C6—C1—S1—O2	6.42 (18)
C8—C9—C10—C11	0.1 (4)	C2—C1—S1—O2	−167.56 (16)
Cl1—C9—C10—C11	179.6 (2)	C6—C1—S1—N1	−108.71 (17)
C9—C10—C11—C12	0.6 (4)	C2—C1—S1—N1	77.30 (18)

### Hydrogen-bond geometry (Å, º)

**Table:**

*D*—H···*A*	*D*—H	H···*A*	*D*···*A*	*D*—H···*A*
N1—H1*N*···O3	0.82 (2)	2.33 (2)	2.864 (2)	123 (2)
N1—H1*N*···O2^i^	0.82 (2)	2.33 (2)	3.061 (2)	148 (2)

Symmetry code: (i) −*x*, *y*+1/2, −*z*+1/2.
